# On Molecular Dynamics and Charge Transport in a Flexible Epoxy Resin Network

**DOI:** 10.3390/ma15186413

**Published:** 2022-09-15

**Authors:** Orestis Vryonis, Alun S. Vaughan, Thomas Andritsch, Peter H. F. Morshuis, Aurore Claverie

**Affiliations:** 1 The Tony Davies High Voltage Laboratory, Department of Electronics and Computer Science, Faculty of Engineering and Physical Sciences, University of Southampton, Southampton SO17 1BJ, UK; 2 Solid Dielectric Solutions Leiden, 2311SG Leiden, The Netherlands; 3 Single Buoy Moorings Inc., 1723 Marly, Switzerland

**Keywords:** flexible epoxy, molecular dynamics, dielectric relaxations, charge transport

## Abstract

An epoxy based on diglycidyl ether of bisphenol A was reacted with a long-chain poly(oxypropylene diamine) hardener in the presence of an accelerator, resulting in a flexible epoxy network. Tensile properties were tested as a function of accelerator concentration. All systems exhibited high levels of extensibility, with strain at failure values in excess of 65%. Molecular dynamics in a formulation containing 10 phr of accelerator were then examined using dielectric spectroscopy over the temperature range of 103–433 K. At low temperatures, a molecular relaxation process (γ relaxation) was observed and shown to conform well to both the Arrhenius equation and activated tunnelling. A stronger relaxation appeared (203–303 K) just before the onset of charge transport, which dominated the behaviour at higher temperatures. The former takes an unusual bimodal form, which we consider a result of overlapping β and α relaxations, consequently termed αβ mode. Analysis of this mechanism revealed a Vogel–Fulcher–Tammann (VFT) behaviour. The temperature-dependent DC conductivity, σ_DC_ (deduced from the low-frequency charge transport contribution to *ε_r_*″), also revealed VFT behaviour with an onset statistically equivalent to that of the αβ mode, therefore suggesting that charge transport, at this temperature regime, is strongly affiliated with cooperative molecular motion.

## 1. Introduction

Epoxy resins represent a versatile class of thermosetting polymers that are used in many applications, such as the matrix material in high-performance structural composites, in electronic systems, and as a component of high voltage insulation. These systems commonly involve the reaction between an epoxy prepolymer containing two epoxide groups, such as diglycidyl ether of bisphenol A (DGEBA) and a suitable anhydride or amine-containing compound (hardener) [1]. Considering, for example, a polyoxypropylene diamine, then each of the two terminal primary amine groups is able to react with two epoxide groups to give a network topology where the separation between nodes is a reflection of the molecular structure of the epoxy prepolymer and the molecular structure of the hardener [2,3]. The resulting materials are generally brittle solids, characterised by a low elongation to break. However, in many applications, toughness and ductility are required and, consequently, numerous strategies have been devised in order to modify the mechanical behaviour of such systems. These include, for example, the following: inclusion of an additional epoxy-containing compound, commonly referred to as a reactive diluent; modification of the structure of the epoxy prepolymer; modification of the structure of the hardener, to introduce additional molecular flexibility into the final network structure.

The addition of so-called reactive diluents has long been used to tailor the processability or mechanical behaviour of epoxy resins, and a wide number of compounds have been examined in this respect [4]. Examples include 1,4-butandiol diglycidyl ether [5] and furfuryl glycidyl ether [6]. Huang and Nie [7] described a modified resin (MR) prepared by dissolving DGEBA in acetone and then adding hydrazine hydrate. Mechanical characterisation of the MR with DGEBA cured with the commercial polyoxypropylene diamine hardener (Jeffamine D-230) revealed a monotonic increase in elongation to break with increasing MR content. Rather than affecting the chain length of the prepolymer, as above, an alternative approach is to modify the epoxy through grafting some appropriate side chains. In addition to modifying the chain packing and, thereby, the thermos-mechanical behaviour of the final thermoset, this also offers the potential of introducing additional functionality. In one embodiment, a flexible siloxane chain containing a phosphaphenanthrene structure (termed KHDOPO) was grafted onto an epoxy prepolymer with the aim of improving flame retardancy. Of relevance here, the resulting KHDOPO-grafted DGEBA-based systems exhibited improved toughness and a moderate increase in elongation to break [8]. Elsewhere, a dodecyl segment was grafted onto an epoxy prepolymer using a thiol-ene click reaction [9]. The resulting systems exhibited a maximum elongation at break, approaching an order of magnitude increase compared with that seen in the unmodified, reference epoxy system. An alternative attempt to modify the elastic response of a DGEBA-based epoxy resin was described by Liu et al. [10], who copolymerised DGEBA with diglycidyl ether of diethylene glycol (DGEG); the inclusion of DGEG led to an increase in the elongation at break. In all of the systems considered, a single *T_g_* was detected by DSC, indicating a homogeneous single-phase structure; the incorporation of DGEG led to only a small reduction in *T_g_*.

Consider now the effect of modifying the hardener system rather than the epoxy prepolymer. Yang et al. [11] considered the curing behaviour of a DGEBA epoxy prepolymer with a range of hardener systems composed of diethyl toluene diamine and two polyoxypropylene diamines, Jeffamine D-230 and Jeffamine D-400. The stress-strain behaviour was found to vary with hardener composition, with the elongation to break increasing monotonically as the diethyl toluene diamine was substituted with either diamine. The presence of a single *T_g_* was taken to indicate that all systems had a homogeneous phase structure. Elsewhere, DGEBA cured with mixtures of an in-house synthesised di-amino terminated polyurethane and the longer polyoxypropylene diamine Jeffamine D-2000 have been examined with respect to their dynamic mechanical characteristics. A progressive decrease in *T_g_* was reported with increasing D-2000 content, along with an increase in the value of tan *δ* associated with the *T_g_*. Furthermore, the temperature dependence of tan *δ* indicated the presence of two phases in certain systems, which was related to the formation of discrete D-2000 rich inclusions [12].

It is apparent from the above examples that the influence of network topology on a wide range of mechanical parameters has been considered. Nevertheless, in electronic and electrical applications, the influence of the electric field on the system is also of great importance; this topic has, however, received relatively little attention. The influence of reactive diluents on the breakdown behaviour and dielectric response of DGEBA-based epoxies has been reported in previous studies [13,14,15], but, in all cases, the focus was on the inclusion of relatively low levels of diluents and the behaviour of the resulting systems in the glassy state. Elsewhere, a number of studies have considered the electrical behaviour of the commercial epoxy resin Araldite CY1311 [13,14,15,16,17], (a modified version of Araldite CY1301 containing added plasticiser). While that work revealed significant differences between the dielectric response and electrical tree growth behaviour of CY1301 (at the rubbery state) and CY1311 (at the glassy state), the direct influence of the plasticiser is unclear. Finally, we are aware of very few investigations concerning molecular relaxation processes in systems that can be considered flexible epoxy resins. For example, Feng et al. [18] considered a material system described as being based upon an epoxy resin, a “flexibilizer” added up to 50%, a methyl hexahydrophthalic anhydride, and N-N-dimthylbenzylamine as the accelerator. Unfortunately, the composition of these systems is poorly defined, rendering critical analysis of the molecular origin of the reported effects impossible. Nevertheless, the results reported indicate that *T_g_*, as measured by DSC, decreases monotonically with an increasing concentration of the flexible epoxy resin. Above the relevant *T_g_*, all systems revealed evidence of pronounced molecular relaxation processes, together with a strong charge transport contribution to the dielectric losses. Furthermore, the authors concluded that the DC conductivity (σ_DC_) could be well described by the empirical Vogel–Fulcher–Tammann (VFT) equation [19]. No other relaxation processes, or their origins, were presented, therefore leaving that area rather unexplored.

The work reported here set out to design a mechanically flexible DGEBA-based epoxy resin with well-defined chemical characteristics and molecular structure; characterise the dielectric molecular dynamics, as well as the charge transport behaviour of this system; establish a novel “structure-property” connection between flexible molecular networks and their dielectric spectra, through corroboration of the acquired experimental data with relevant studies published in the literature.

## 2. Materials and Methods

Samples were produced using a DGEBA prepolymer with an epoxide equivalent molar mass (EEW) of 172–176 g/mol (DER 332, obtained from Sigma Aldrich, Gillingham, UK) and a long-chain polyoxypropylene diamine hardener (Jeffamine D-4000 obtained from Huntsman, Everberg, Belgium), which has an amine hydrogen equivalent molar mass of 1000 g/mol. Since we, initially, opted for a well-defined molecular structure, we attempted curing the abovementioned components without the addition of any other moieties, only to find that the reaction kinetics were not sufficiently “agitated” to promote crosslinking even several weeks upon mixing (mixture was still in liquid phase). Therefore, to promote curing, a commercial accelerator (Accelerator 400 obtained from Huntsman, Everberg, Belgium) was also added; this has an amine hydrogen equivalent molar mass of 145 g/mol. Amine curing of epoxy resins involves comparatively simple chemistry and, as such, the stoichiometric ratio of prepolymer to hardener (plus accelerator) was chosen to correspond to the theoretical ideal. That is, one epoxide group per amine hydrogen, where the total number of amine hydrogen is the sum of those supplied by both the hardener and the accelerator. This is in line with Huntsman’s recommendations. In order to align with the notion of “least amount” of molecular variations possible within the molecular network we investigated a range of accelerator contents with the intention of attaining a fully crosslinked system within the suggested (by Huntsman) curing/post-curing duration that incorporates the least amount of accelerator. Therefore, the accelerator contents spanned between 5 and 20 phr (parts accelerator per 100 parts resin), as seen in Table 1; only those incorporating contents above 10 phr fully cured into a solid, while the one with 20 phr displayed a rather sticky surface texture, yellow colour, and difficulties in handling.

The required samples were prepared as follows. First, the epoxy prepolymer was heated at 323 K to reduce its viscosity. In parallel, the accelerator was mixed with the hardener before being added to the epoxy prepolymer. The resulting system was thoroughly mixed to produce a homogeneous liquid, degassed in a vacuum chamber for 20 min, and then transferred into the required moulds for curing. The curing process included an initial step at 353 K for 2 h, followed by a second step at 398 K for 3 h; these parameters were derived from an optimisation study (where the duration of curing/post-curing was optimised with respect to DSC-derived *T_g_* values) and are in line with the manufacturer’s suggestions. Finally, the samples were left inside the oven gradually to reach room temperature, before being demoulded and stored in a vacuum desiccator until required. For mechanical measurements, dumbbell-shaped samples with a thickness of 4 mm were cast, according to ASTM D638-02A (ASTM International, PA, West Conshohocken, PA, USA). For dielectric measurements, the resin mixture was vacuum transferred into metallic-plate moulds, separated by a Melinex (Profoil Systems Limited, St. Albans, UK) film spacer ~200 μm in thickness. 

Mechanical stress/strain data were obtained at room temperature using a Tinius Olsen H25KS tensometer (Salfords, UK) at a strain rate of 50 mm/min until sample failure. Dielectric data were acquired using a Schlumberger SI 1260 impedance/phase gain analyser, connected to a Solartron 1296 dielectric interface system; a Janis Research STVP-200-XG cryostat sample holder system (Farnborough, UK) was used to vary the sample temperature. Samples were routinely sputter coated with gold to give opposing electrodes, 20 mm in diameter. Data were acquired from 103–433 K in 10 K steps and, at each temperature, a frequency sweep was conducted from 10^−1^ to 10^4^ Hz using a *V_RMS_* AC voltage of 7 V.

## 3. Results

### 3.1. Tensile Behaviour

**Figure 1:**
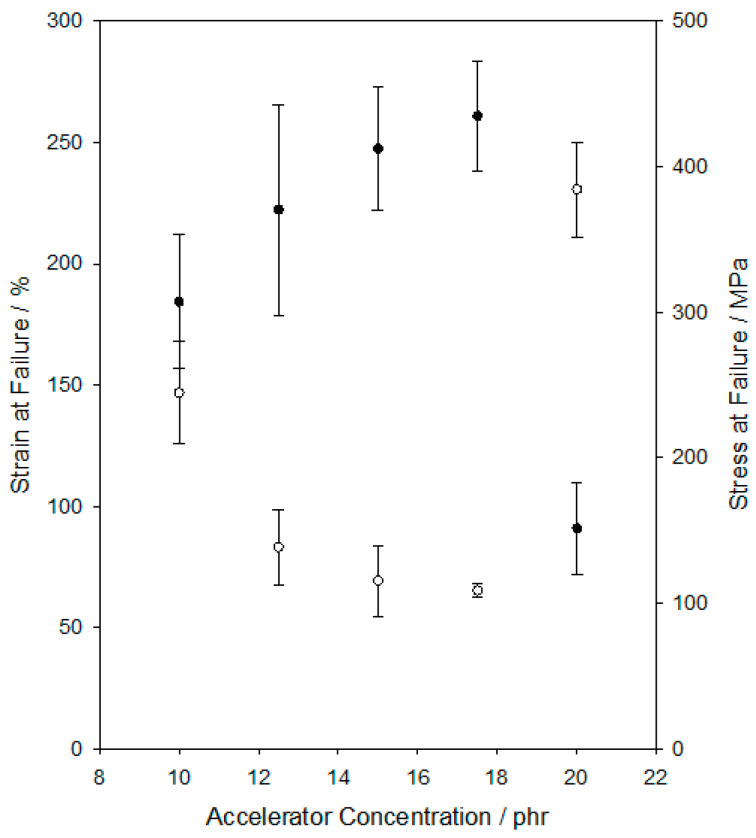
Plots showing the effect of accelerator concentration on strain at failure (ο) and stress at failure (●).

Stress/strain data were obtained from a range of systems containing different accelerator concentrations and derived data are presented in Figure 1. Since the topic of interest here concerns the flexibility of the various systems, the effect of accelerator concentration on the strain at failure and the stress at failure is shown. From Figure 1, it is an event that, for the systems studied, the strain at failure falls monotonically over the accelerator composition range of 10–17.5 phr, before increasing markedly in the system formulated with 20 phr of the accelerator. The stress at failure varies in an inverse manner, increasing progressively from 10–17.5 phr before falling in the system containing 20 phr of the accelerator.

From Figure 1, it is evident that all of the systems studied exhibit strain at failure values in excess of 65%, with a maximum value of 231 ± 20% being seen in the system formulated with 20 phr of the accelerator. As mentioned earlier, though, the latter sample was quite challenging in handling and displayed different physical characteristics compared with the rest, indicating that the system is strongly influenced by the accelerator phase, potentially affecting even the crosslinking kinetics. Use of a similar, or even higher, accelerator concentration is not recommended.

As described above, a range of strategies have previously been investigated in connection with the preparation of flexible epoxy resins, with strain at failure values up to about 80% being reported in the cited examples [7,8,9,10,11]. While this figure is in no way meant to represent an upper bound, it does, nevertheless, provide a useful benchmark for the data shown in Figure 1—the systems synthesised in our study can be considered as highly flexible epoxy resins, with levels of flexibility at least commensurate with those previously reported in the literature. As such, the strategy described here of employing a long amine-terminated polyoxypropylene chain together with an amine-based accelerator is successful in generating a flexible DGEBA-based system for study.

In view of the aims of this work, the system containing 10 phr of the accelerator was chosen for further study—the minimum concentration necessary to provide the required, practical, flexible systems (strain at failure: 147 ± 20%).

### 3.2. Dielectric Response

**Figure 2:**
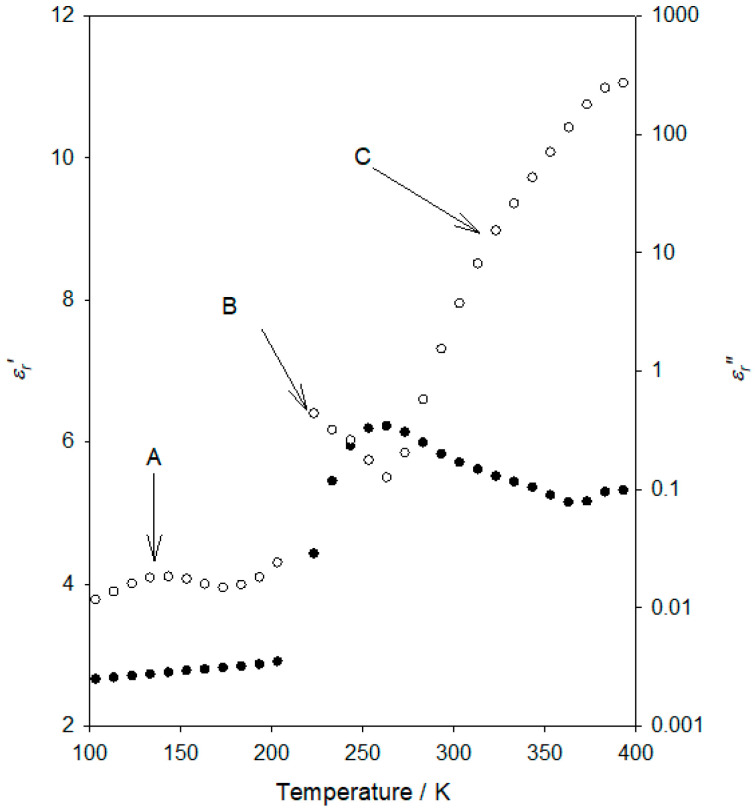
Temperature dependence of the real part of the relative permittivity, *ε_r_*′ (●, left hand vertical axis) and the imaginary part of the relative permittivity, *ε_r_*″ (ο, right hand vertical axis), measured at a frequency of 10 Hz.

Figure 2 provides an overview of the dielectric response of our selected flexible epoxy resin formulation at an applied frequency of 10 Hz. In this figure, the variation of the real (*ε_r_*′) and imaginary (*ε_r_*″) parts of the relative permittivity is depicted against temperature.

Consider first the temperature variation of the real part of the relative permittivity shown in Figure 2, in which a progressive increase in *ε_r_*′ is seen at low temperatures, followed by a pronounced, sharp, increase, and finally, a gradual decrease, in *ε_r_*′, as the temperature increases further. From the imaginary permittivity data, it is evident that the behaviour of the system can be considered in terms of three processes, which are arrowed and labelled “A” (~140 K), “B” (~220 K) and “C” (temperatures above ~260 K), in order of increasing temperature. Process “B” corresponds to the sharp *ε_r_*′ increase.

Before examining these processes in turn, it is worth considering the general form of these plots. Kourkoutsaki et al. [20] described a study of polymer dynamics in rubbery epoxy networks/polyhedral oligomeric silsesquioxanes (POSS) nanocomposites that were formulated using DGEBA as the epoxy prepolymer and Jeffamine D-2000 as the hardener. As such, that study has close parallels with the investigation described here, albeit a hardener of relatively lower molecular weight, therefore of a slightly “stiffer” nature. In the case of the system containing no POSS, they reported the following four processes: three dielectric relaxation peaks in the temperature range of 140–270 K, together with higher temperature features associated with the normal mode relaxation and charge transport. In line with conventions, these workers termed the weak relaxation seen at about 140 K (10 Hz) as the γ mode, the relaxation at about 190 K as β, and the strong process at ~240 K was termed the α mode. This final attribution was consistent with a DSC-derived *T_g_* value. These workers also related the reduction in *ε_r_*′ above the α mode to processes that are “typically observed in amorphous polymers and glass-forming liquids at temperatures higher than *T_g_*”. Similar effects in *ε_r_*′, at temperatures just above the α relaxation/*T_g_*, have been observed elsewhere in DGEBA/ethylenediamine systems [21] and poly(vinyl acetate) [22] systems. In short, the dielectric data shown in Figure 2 are broadly consistent with relevant published data.

We consequently concur with the following:

(1) The dielectric spectra shown in Figure 2 are in line with comparable published work;
(2) The *T_g_* of the flexible epoxy resin featured here is around the strong process labelled “B”, about −55 °C (~220 K), which is in good agreement with studies featuring equivalent chemistries (Jeffamine D-4000) [23,24];
(3) The epoxy resin in [20] (cured with the “stiffer” Jeffamine D-2000) shows a *T_g_* ~ 20 degrees higher, as well as one additional apparent relaxation peak than the epoxy in the present work.

### 3.3. The Lower Temperature Dielectric Relaxation (γ mode)

**Figure 3:**
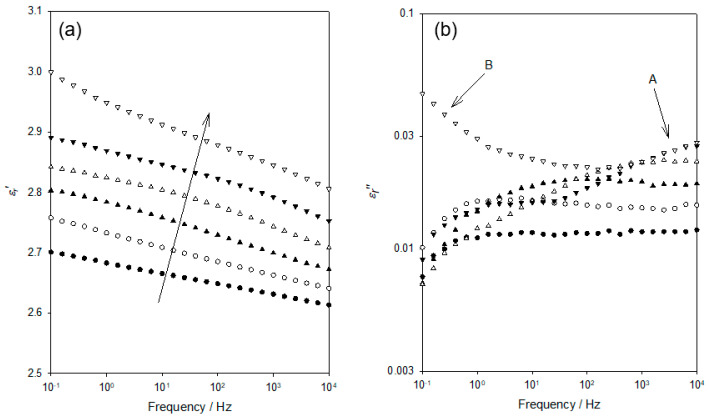
Dielectric data acquired at temperatures of 103 K (●), 123 K (ο), 143 K (▲), 163 K (△), 183 K (▼), and 203 K (▽): (**a**) frequency dependence of the real part of the relative permittivity, *ε_r_*′; (**b**) frequency dependence of the imaginary part of the relative permittivity, *ε_r_*″.

Figure 3 shows the frequency dependence of *ε_r_*′ and *ε_r_*″ within the temperature range relevant to the process labelled “A” in Figure 2. Although data were acquired at intervals of 10 K, only six data sets are shown here, for clarity. Considering, first, the *ε_r_*′ (Figure 3a), the data acquired at 103 K evince a progressive increase in permittivity values as the frequency is reduced and the temperature increased. Figure 3b shows the frequency dependence of *ε_r_*″ across the same range of temperatures. At 103 K and ascending frequency, the *ε_r_*″ appears first to slightly increase (0.1–1 Hz) and then to remain constant up to a frequency of 10^4^ Hz; no distinct peak is evident. While the data acquired at 123 K appear to take a comparable form, albeit with *ε_r_*″ increased significantly across the complete frequency range, examination of the numerical data reveals the presence of a very weak maximum at ~6 Hz. At higher temperatures, the associated values of *ε_r_*″ continue to increase, with the weak peak maximum occurring, for example, at ~120 Hz at 143 K. At temperatures greater than 163 K, no peak is evident, with the peak maximum seemingly lying beyond our accessible frequency range.

**Figure 4:**
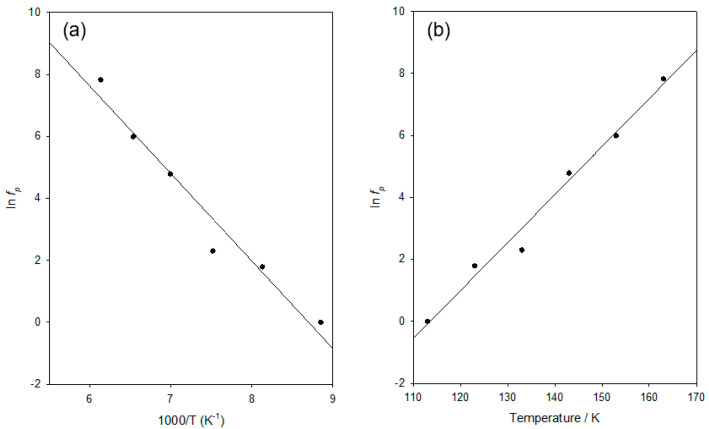
Temperature, *T*, dependence of the estimated dielectric γ relaxation peak frequency, *f_p_*, plotted according to the: (**a**) Arrhenius equation and (**b**) thermally activated tunnelling.

In view of the temperatures and frequencies involved in Figure 3, we suggest that the dielectric processes involved align with the γ relaxation described in conventional epoxy systems. This process has previously been related to a range of different structural elements, which have been reviewed and shown, in our previous study [25] to be related either to unreacted chain ends and/or (depending on what is applicable) main chain units, including sequences of methylene groups and polar ether linkages. In the latter work, γ_1_ was related to the main chain process, γ_2_ was associated with unreacted epoxide units. The characteristic frequency associated with γ_1_ was shown to vary slowly with temperature, while that of γ_2_ varied much more markedly. From Figure 3b, it is evident that, in our DGEBA/D-4000 system, the form of the dielectric γ relaxation is highly temperature dependent and that the associated form of any peak is poorly defined and variable, such that a rigorous analysis using, for example, the Havriliak-Negami (HN) formalism [26] or the time/temperature superposition principle to generate a relaxation master curve is questionable. Nevertheless, it is possible to estimate from the numerical data the frequency, *f_p_*, corresponding to the local maximum in *ε_r_*″ in data sets covering a reasonable temperature range (113–163 K) and, as such, it is pertinent to consider the implications of this (see Figure 4).

In Figure 4a, the temperature dependence of the resulting estimated *f_p_* values is represented in a form relevant to the Arrhenius equation, whereby ln(*f_p_*) is plotted against 1/*T* to give a linear form. That is as follows:

(1) $$f_{p}=A_{0}exp\left(-\frac{E_{a}}{kT}\right)$$

where *A*_0_ is a constant with dimensions of s^−1^ (i.e., *A*_0_ is equivalent to a frequency) and k is the Boltzmann constant. From this, it is evident that the data conform well to the Arrhenius equation (*R*^2^ value of 0.962). However, it is evident from the residuals that these are not randomly distributed around the best fit line and, consequently, as recently described in our latest work [27], the same data set was also analysed in terms of the activated tunnelling model of Hill and Dissado [28]. In short, the approach considers quantum mechanical tunnelling through a potential barrier of height Δ between potential energy minima separated by a distance *d*_0_. That is, while the Arrhenius equation considers thermal excitation over a potential energy barrier, activated tunnelling considers that the transition between states on either side of a potential energy barrier may occur by thermal excitation to a level below the top of the barrier, combined by tunnelling through the barrier. For this, the optimum relaxation rate, *f_p_*, can be written as follows:

(2) $$f_{p}=f_{t}exp\left(\frac{2}{15}A^{2}d_{0}^{2}kT\right)$$

where *A* and *f_t_* are constants. Thus, plotting ln(*f_p_*) against *T* would lead to a linear dependence, if the theory were applicable. Figure 4b shows such a plot, from which good linearity is evident (*R*^2^ values of 0.983). Furthermore, in this case, the residuals are randomly distributed, indicating that while both approaches describe the behaviour well, the data conform better to the activated tunnelling equation than to the Arrhenius equation.

Parameters derived from both approaches are presented in Table 2. At first sight, the behaviour presented in Figure 4 may appear contradictory in that it suggests, from a mathematical perspective, that ln(*f_p_*) varies in a close to the linear manner (*R*^2^ approaching 1) when plotted against both 1/*T* and *T*. Furthermore, the underlying physics involved in the Arrhenius behaviour and activated tunnelling is also rather different. To address this, it is necessary to examine the concept of activated tunnelling in more detail and, specifically, to consider the temperature range over which the tunnelling aspect has an appreciable mechanistic influence. For the tunnelling process to be significant, thermal activation must occur to an excited state below the top of the potential barrier as follows: that is, the process will only be significant at low temperatures. Previously, Hill and Dissado [28] have shown that the corresponding maximum temperature, *T_max_*, of applicability can be written as follows:

(3) Tmax=7.5∆12Ad0k

albeit that the form of this equation is dependent on the precise shape of the potential barrier, such that the resulting value of *T_max_* should only be considered as an estimate. Nevertheless, the substitution of values from Table 2 into the above leads to a value of *T_max_* of 155 K.

Furthermore, the frequency corresponding to the pre-exponent Arrhenius parameter, *A*_0_, is approaching the THz regime and therefore is physically reasonable; that is, it is of a comparable order to the vibrational frequency of the relevant molecular structural units. In summary, the above results suggest that, over the temperature range considered, the behaviour of the system is moving away from activated tunnelling towards an Arrhenius behaviour. Similar behaviour was reported in [27], particularly in systems that contained moieties that enhanced molecular mobility. We, therefore, concur with the above suggestion that, in the context of Figure 4, “pure thermal activation, as described by the Arrhenius equation is […] just a manifestation of activated tunnelling above some material dependent temperature”. Furthermore, the above analysis demonstrates that the data shown in Figure 3 are quantitatively consistent with the characteristics of the dielectric γ relaxation in other DGEBA-based epoxy systems. 

### 3.4. The Higher Temperature Dielectric Relaxation (αβ mode)

**Figure 5:**
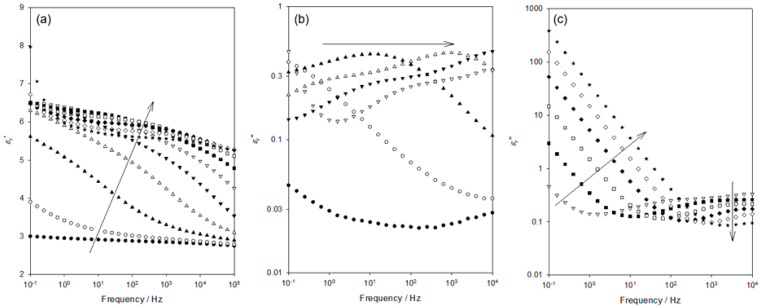
Dielectric spectra acquired at temperatures of 203 K (●), 213 K (ο), 223 K (▲), 233 K (△), 243 K (▼), 253 K (▽), 263 K (■); 273 K (☐), 283 K (◆), 293 K (◇), and 303 K (∗). Frequency dependence of the: (**a**) real part of the relative permittivity, *ε_r_*′; imaginary part of the relative permittivity, *ε_r_*″ at: (**b**) 203–253 K and (**c**) 253–303 K.

The data set shown in Figure 3 that was acquired at 203 K includes a pronounced increase in *ε_r_*″ at low frequencies, which is arrowed and indicated “B”. This corresponds to the molecular relaxation process similarly labelled “B” in Figure 2. Figure 5 contains dielectric data acquired over the temperature range of 203–303 K as follows: the temperature dependence of *ε_r_*′ over this complete temperature range is shown in Figure 5a while, for clarity, the corresponding *ε_r_*″ data are shown in Figure 5b (203–253 K) and Figure 5c (253–303 K). Consider, first, the former temperature range. From Figure 5a, it is evident that *ε_r_*′ increases markedly over the above temperature range. This increase is a consequence of the strong dielectric relaxation process that moves to progressively higher frequencies as the temperature is increased. From Figure 5b, the following two features are worthy of note: first, the shape and strength of this peak are largely invariant with temperature; second, the form of the peak differs greatly from the ideal Debye relaxation or variants of this such as the HN formalism. Rather, the form of the peak is suggestive of the main process supplemented by an additional shoulder at lower frequencies (see, for example, the data set acquired at 233 K).

**Figure 6:**
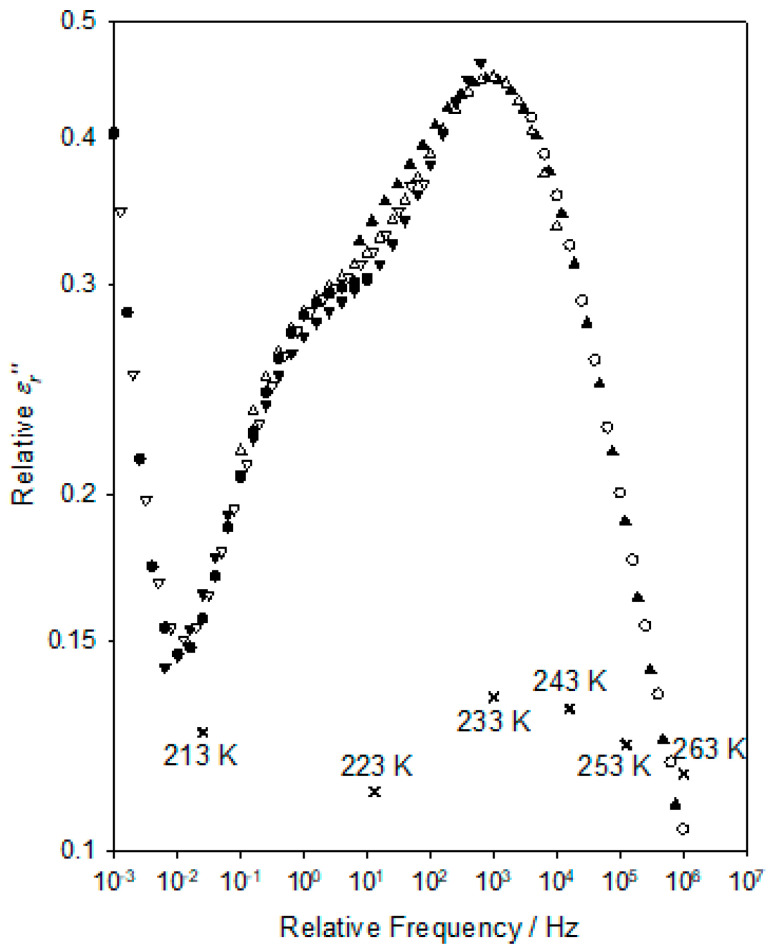
Dielectric relaxation master curve for the high temperature process. Data acquired at: 213 K (ο), 223 K (▲), 233 K (△), 243 K (▼), 253 K (▽), and 263 K (■).

This latter feature becomes more evident when the data sets are combined into a relaxation master curve, as shown in Figure 6; the corresponding relative shifts in frequency and *ε_r_*″ values are indicated in this by the *X* symbols. The shift factors and consequent relative peak frequency, *f_p_*, values generated in producing Figure 6 facilitate a quantitative analysis of the temperature dependence of the process and, in this respect, two approaches may be relevant. The low-temperature γ relaxation is typically reported in conventional DGEBA-based systems to be followed at intermediate temperatures by the β relaxation, which, in amine-cured systems, is commonly associated with the local motion of the hydroxyl-ether groups formed during curing [3]. At yet higher temperatures, the α relaxation is seen, which is related to the motion of larger chain segments that occur around *T_g_*, the temperature dependence of which is widely reported to conform to the VFT equation [29,30] that is the following:

(4) $$f_{p}=A_{VFT\alpha }exp\left(\frac{B_{\alpha }}{T-T_{VFT\alpha }}\right)$$

where *A_VFT_*_α_ *B*_α_ and *T_VFT_*_α_ are empirical, material-dependent parameters relating to the dielectric α process. However, *T_VFT_*_α_ is typically found to lie some 50 K below *T_g_* [31]. A comparison of the temperature dependence of the relative frequency values with the Arrhenius equation demonstrates that the dielectric process shown in Figure 6 does not conform to the Arrhenius equation (results not shown for the sake of brevity). A number of methodologies are possible in fitting the experimental data to the VFT equation, including the direct approach of treating *A_VFT_*_α_, *B*_α_ and *T_VFT_*_α_ as free parameters and fitting *f_p_* to *T* using appropriate fitting software. However, such an approach is problematic in this case for the following reason: The reliability of the fit and the derived parameters are strongly influenced by the uncertainties in the input data and the local curvature of the objective function hyperplane. Specifically, if the latter in the vicinity of its global minimum is low, then a large number of potential solutions exist that are of close to equal statistical validity. Essentially, if *T_VFT_*_α_ is changed, then the VFT *A_VFT_*_α_ and *B*_α_ parameters can be adjusted to compensate such that another, alternative solution arises with a comparable value of *R*^2^. When the goodness of fit to these different solutions is comparable (bearing in mind the uncertainties in the experimental input data), then it is impossible to choose between them on the grounds of statistical fit alone. However, the above fails to consider the form of the residuals, which can easily be gauged visually. For this reason, the above purely statistical approach was not followed; rather, values of *T_VFT_*_α_ were sequentially chosen and ln(*f_p_*) plotted against 1/(*T* − *T_VFT_*_α_). In this way, it is possible to consider the goodness of fit in terms of the resulting dependence of both *R*^2^ and the form of the residuals on the chosen value of *T_VFT_*_α_.

**Figure 7:**
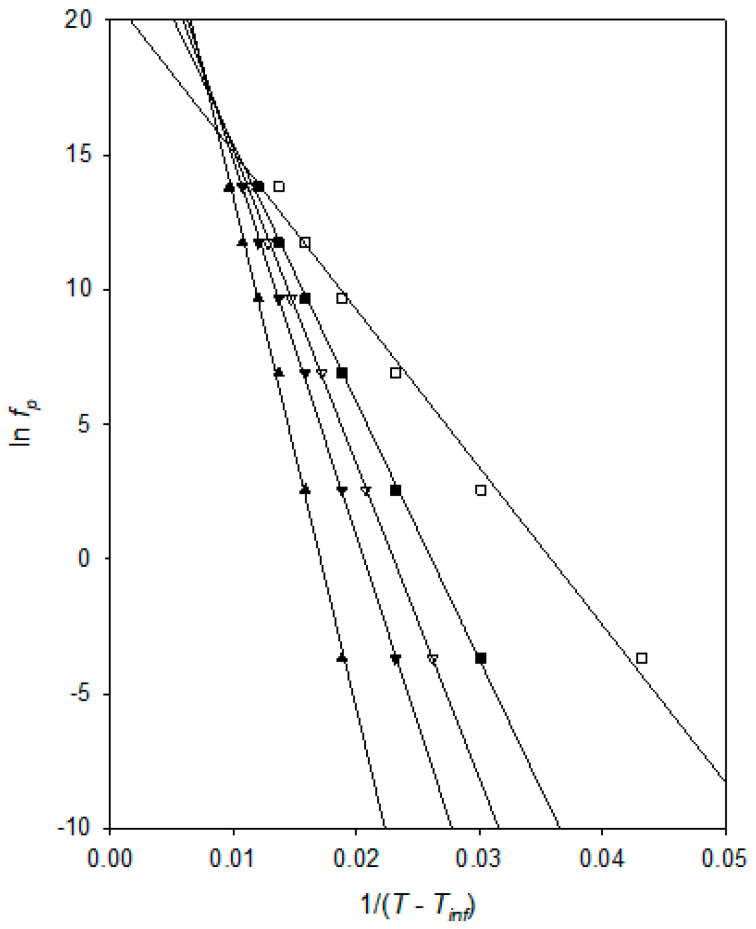
Analysis of the temperature dependence of the higher temperature dielectric relaxation of the rubbery epoxy system: *T_VFT_*_α_ = 160 K ▲; *T_VFT_*_α_ = 170 K ▼; *T_VFT_*_α_ = 175 K ▽; *T_VFT_*_α_ = 180 K ■; *T_VFT_*_α_ = 190 K ☐.

Figure 7 presents a VFT analysis of the peak frequency, *f_p_*, values derived from Figure 6. In this, the various fit lines shown were obtained by assuming the indicated value for *T_VFT_*_α_, from which, it is evident that all of these correspond to good fits to the experimental data, with *R*^2^ values varying from 0.9975 (*T_VFT_*_α_ = 160 K) through 0.9996 (*T_VFT_*_α_ = 175 K) to 0.9905 (*T_VFT_*_α_ = 190 K). Furthermore, a comparison of each set of data points with the relevant fit line indicates that the form of the residuals varies systematically with the chosen value of *T_VFT_*_α_. For example, in the case of *T_VFT_*_α_ = 190 K, the data points fall, not along a straight line, but rather, take the form of a concave curve, (the data points tend to lie *above* the fit line at high and low temperatures, but *below* the fit line at intermediate values). Conversely, although not as obvious, the data points lie on a convex curve when *T_VFT_*_α_ = 160 K. In summary, both indicators (*R*^2^ and residuals) point to an optimum value of *T_VFT_*_α_ = 175 ± 5K, suggesting a *T_g_* value around ~225 K, which is in excellent agreement with the assumptions made above (suggesting the *T_g_* is located around process “B” (~220 K) and the literature reporting a value of about −55 °C (~220 K)). [23,24] As such, the above *T_VFT_*_α_ value is physically credible.

It is evident that at temperatures above those associated with the dielectric γ relaxation, the DGEBA/D-4000 system exhibits not the conventional, distinct β and α processes but, rather, just a singular, bimodal, process, the temperature dependence of which aligns with that normally associated with the α relaxation. This form of behaviour is, therefore, the explanation for the “absence” of a relaxation peak in DGEBA/D-4000, in Figure 2, as compared to the equivalent plot in DGEBA/D-2000, in [20]. Such an effect is unusual and requires some discussion. 

The sub-*T_g_* relaxation behaviour of epoxy-based systems has been reported by many workers but, here, we will focus on the work of Mangion and Johari [32,33], which provides particularly useful insights into the origin of the effects we report. In summary, these workers considered the dielectric relaxation behaviour of DBEBA cured using two amine-based hardeners, namely, with diaminodiphenyl methane (DDM) and diaminodiphenyl sulfone (DDS), and in both systems, they analysed changes in the dielectric relaxation processes during curing. Focusing, first, on the γ and β relaxations, it is reported that, as curing progresses, “the strength of the γ process decreases and reaches a limiting value, while that of the β process initially increases, reaches a maximum value, and then decreases” [32]. As is usual, these workers associated the β relaxation with hydroxyl-ether segments formed as a consequence of crosslinking reactions between epoxide and amine groups, the concentration of which increases as curing progresses, thereby explaining the initial increase in the associated relaxation strength. Consequently, as curing proceeds further, the hydroxyl-ether group’s concentration is more than offset by constraints on the molecular motion within the glassy structure, thereby the strength of the β relaxation slightly decreases. Furthermore, as curing proceeds, the temperature of the α relaxation increases, such that it becomes increasingly separated from the β process [32,33].

Evidently, the system characterised in our study was formulated using the theoretical optimum stoichiometry and was fully cured. Certainly, the data presented in Figure 3 are consistent with this, based upon previous work [25], in that there is no evidence of γ_2_ mode that would imply unreacted end-groups. As such, when considering the behaviour of any β relaxation in the context of the work of Mangion and Johari [32,33], the major difference between systems cured with DDM or DDS and D-4000 is the molecular architecture of the curing agent. Specifically, when fully cured using DDM or DDS, the molecular network of a DGEBA-based epoxy is highly constrained, leading to *T_g_* values in excess of 370 K [32,34]; only slight variations between the β relaxations of the two different systems were observed and assigned to the structures of the respective hardeners, which resulted in local loose packing of chain segments. Conversely, the use of D-4000 leads to a marked increase in the contour length between network nodes and a commensurate reduction in molecular constraints.

In general, the hardener’s structure, whether it be amine-based or anhydride, has been shown in many cases to have a great impact on the α relaxation due to associated variations in the *T_g_* and a lesser impact on the β relaxation. For instance, one of the very first studies to consider the hardener’s profile in dynamic response was by Shito et al. [35], where the dielectric and mechanical dynamic spectra of epoxy resins cured with eight different anhydrides revealed the following two relaxations in the examined temperature range: the α mode (WLF behaviour) and the β mode (Arrhenius behaviour). The former was attributed to “motions of large chain segments which are frozen below the *T_g_*” while the latter was attributed to “smaller segments which are not frozen below the *T_g_* and freeze at a certain lower temperature”. The importance of structural characteristics on the hardener, such as double bonds and ring structures, was pinpointed and shown to have a greater impact on the *T_g_* (therefore α relaxation) of the system.

Dammont et al. [36], in a study involving various prepolymers and (amine-based) hardener structures, showed that the β relaxation displays universal characteristics invariant to prepolymer and hardener structures and was ascribed to the hydroxyl-ether segments of the molecule. It was shown that the location of only the α relaxation (attributed to the *T_g_*) shifts according to the flexibility (aliphatic) or stiffness (aromatic) of the examined molecular structure. Similar findings were reported by an extensive work by Pogani et al. [37,38], where a number of epoxy systems were cured with various ratios of different amine hardeners. The flexibility (aliphatic vs aromatic) of the structure was shown to have a greater impact on the α mode (attributed to *T_g_*) rather than the secondary relaxations.

Following from the literature review reported above, it is this great effect of hardener’s flexible structure on the α mode, significantly shifting it towards the slightly affected β mode which we suggest results in the absence of distinct, well-separated α and β processes. Previously, under conditions where the α and β processes merge, the resulting process has been termed αβ mode [39]. Furthermore, Beiner and Ngai [40] have indicated that secondary relaxations such as the β mode can trigger the many-molecule cooperative α relaxation. That is, in conventional, highly constrained epoxy networks, the α and β do not differ in terms of the dipolar moieties involved but, rather, in terms of the extent to which these are able (due to the network topology) to act cooperatively. In our DGEBA/D-4000 system, these constraints are relaxed to such an extent that α and β processes are, effectively coinciding, and therefore appear merged. In the absence of the local constraints that then differentiate α and β, the behaviour of the resulting αβ process, then exhibits a temperature dependence that conforms to the VFT equation that is, elsewhere, used to characterise only the conventional α process.

### 3.5. Charge Transport

**Figure 8:**
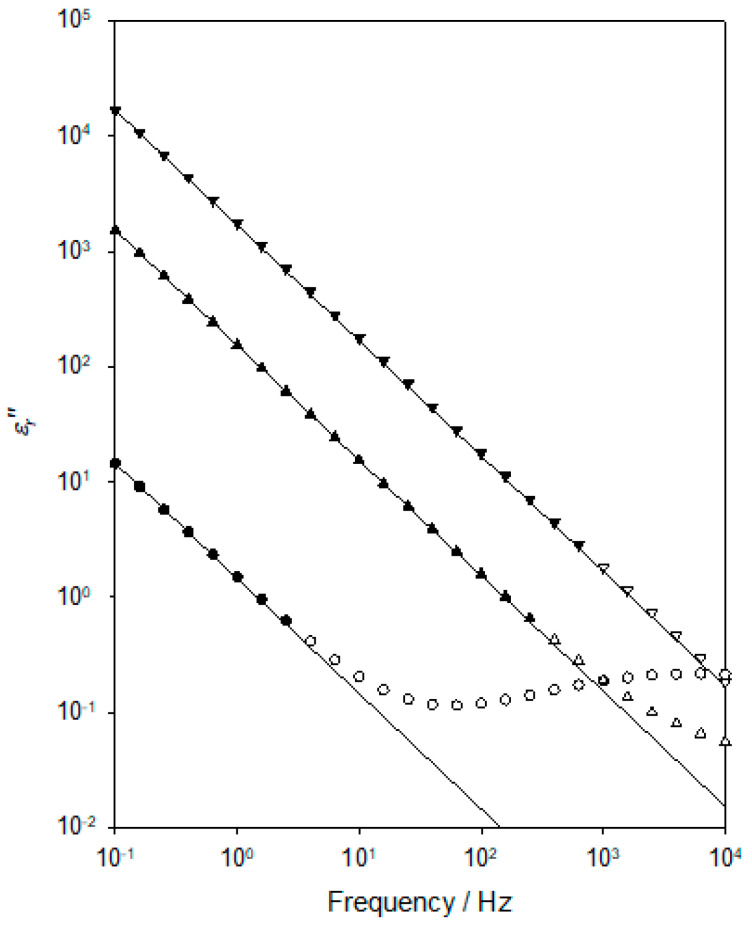
Representative data showing the frequency dependence of *ε_r_*″ at selected temperatures in the range from 273 K to 373 K. Measured *ε_r_*″ data obtained at 273 K (ο); truncated *ε_r_*″ data at 273 K (●) used to determine the indicated fit line. Measured *ε_r_*″ data obtained at 323 K (△); truncated *ε_r_*″ data at 273 K (▲) used to determine the indicated fit line. Measured *ε_r_*″ data obtained at 373 K (▽); truncated *ε_r_*″ data at 273 K (▼) used to determine the indicated fit lines.

Figure 8 contains representative *ε_r_*″ data obtained at three temperatures in the range of 273–303 K. In this figure, for each temperature, the following three plots are shown: the experimentally determined data points (open symbols); a subset of these is used to generate the relevant low-frequency fit line (closed symbols); fit line to the low-frequency data. From theory [41], DC conduction will manifest itself in the dielectric spectrum through a contribution to the imaginary part (slope of −1 in a log-log scale) of the relative permittivity that varies according to the following:

(5) $${\varepsilon_{r}}^{\prime \prime }{}_{DC}=\frac{\sigma_{DC}}{\varepsilon_{0}\omega }$$

where *ε_r_*″*_DC_* is the contribution to the measured value of *ε_r_*″ that arises as a consequence of the DC conductivity, *σ_DC_*, of the system, *ε*_0_ is the permittivity of free space, and *ω* is the angular frequency.

To examine the extent to which the increase in *ε_r_*″ with a decreasing frequency that is evident in Figure 8 is indeed a consequence of DC conduction, two processes were adopted. First, it is possible to estimate the contribution to *ε_r_*″ that arises from polarisation mechanisms using the following form of the Kramers-Kronig equation, a quantity here represented *ε_r_*″*_deriv_* as follows [25]:

(6) $$\varepsilon_{r}{{}^{\prime \prime }}_{deriv}=-\frac{\pi }{2}\frac{\partial \varepsilon_{r}{}^{\prime }\left(\omega \right)}{\partial \ln\omega }$$

**Figure 9:**
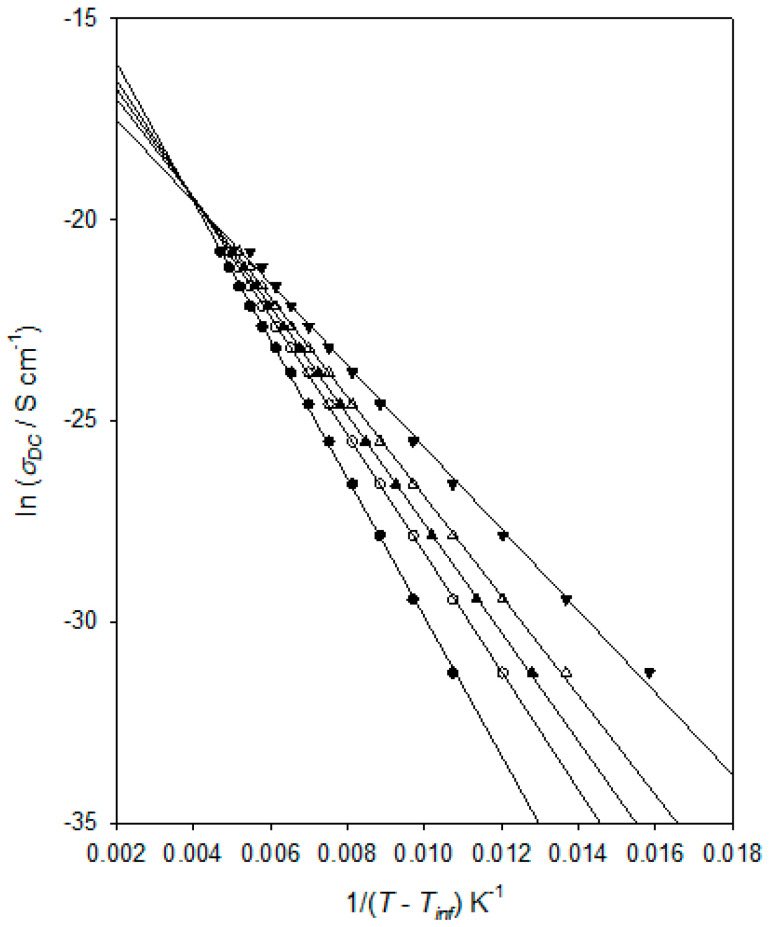
Analysis of the charge transport behaviour of the DGEBA/D-4000 system: *T_VFTσ_* = 160 K (●); *T_VFTσ_* = 170 K (ο); *T_VFTσ_* = 175 K (▲); *T_VFTσ_* = 180 K (△); *T_VFTσ_* = 190 K (▼).

Second, it is possible to examine the experimental data, specifically, to test the hypothesis that the frequency dependence of *ε_r_*″ is primarily a consequence of DC conduction. In this way, we have shown that, over the temperature range of interest, *ε_r_*″ >> *ε_r_″_deriv_* and that *ε_r_*″ ∝ *ω*^−1^: that is, the variation in *ε_r_*″ at low frequencies is indeed, consistent with it predominantly arising from DC conduction. Data of the form shown in Figure 8 were used to determine the temperature dependence of *σ_DC_* for the DGEBA/D-4000 system, and the derived values are plotted in Figure 9 in an equivalent way to that used in Figure 7. That is, according to the following VFT equation:

(7) $$\sigma_{DC}=A_{VFT\sigma }exp\left(\frac{B_{\sigma }}{T-T_{VFT\sigma }}\right)$$

where *A_VFTσ_*, *B_σ_*, and *T_VFTσ_* are empirical, material-dependent parameters relating to the process of DC conduction. As discussed above in connection with the dielectric αβ process, obtaining an optimum value for *T_VFTσ_* can be problematical and, consequently, Figure 9 again shows the effect of varying this parameter. As in the case of Figure 7, all the linear plots shown correspond to good fits to the experimental data, with *R*^2^ values varying from 0.9994 (*T_VFTσ_* = 160 K) through 0.9998 (*T_VFTσ_* = 170 K and 175 K) to 0.9975 (*T_VFTσ_* = 190 K). Obtained values for *R*^2^ continue to decrease outside the *T_VFTσ_* range discussed explicitly above. Furthermore, as in the case of Figure 7, when taking *T_VFTσ_* = 190 K, the data points fall on a concave curve, while taking *T_VFTσ_* = 160 K leads to the data points lying on a convex curve. This curvature becomes increasingly clear as *T_VFTσ_* is varied outside the range shown. For this system, a value *T_VFTσ_* = 173 ± 5 K is therefore determined, whereby the derived values for *T_VFT_*_α_ and *T_VFTσ_* are statistically equivalent. A similar finding was previously reported by Huang et al. [42] for a chemically rather different epoxy system.

The topic of charge transport in epoxy resins has been considered in numerous studies, using different approaches, and a range of possible mechanisms has been suggested. For example, Shimakawa et al. [43] modelled space charge development and conduction in an epoxy resin using a bipolar charge transport model that considered charge trapping, de-trapping, and recombination where the positive charges included both holes and positive ions and the negative charges included both electrons and negative ions. Despite including a number of free parameters in their model, it was found not to be possible to simultaneously reproduce the experimental space charge and the external current data, a result that highlights the complexity of the processes involved. Other studies have similarly invoked a combination of electron, hole, and ionic transport in epoxy resins. For instance, Tian et al. [44,45] interpret their space charge and conductivity measurements in terms of electron and hole transport at temperatures below *T_g_* coupled—at higher temperatures—with the migration of ions. They suggested that above *T_g_*, it is ionic transport that dominates. An alternative mechanism was, however, suggested by Tian and Ohki [46], who considered electrode polarisation and charge transport processes in a DGEBA-based epoxy at temperatures above *T_g_*. This work concluded that ionic transport is mediated by the molecular motion that occurs at temperatures above *T_g_* and that the resulting charge transport contributes to both the measured DC conductivity and, through partial blockage, electrode polarisation. However, the electronic contribution to the DC conductivity was deduced to be one to two orders of magnitude greater than that arising from ions. This study also suggested that the ions may originate from impurities remaining in the system after synthesis and from the ionisation of unreacted epoxy/hardener moieties. As such, the relative importance of electronic and ionic processes is likely to be critically dependent on the chosen reactants and stoichiometry. Indeed, it has also been proposed, in a rather different epoxy-based system, that charge transport above *T_g_* occurs through a bulk quasi–DC transport process involving ionic or electronic transfer between neutral clusters, “leading to an extended charge separation on a percolation structure” [47]. In the system studied, absorbed water and protonic processes were thought to be of potential relevance, and segmental motion was considered to be of importance in mediating the transfer of charge between clusters. Elsewhere, Drakopoulos et al. [48] observed ohmic conduction in their epoxy systems. While this mirrors the behaviour seen in Figure 8 and Figure 9, the assertion of Arrhenius behaviour is very different from the VFT temperature dependence reported in our work and elsewhere [42,49].

From the above discussion, it is apparent that the fundamental mechanism of charge transport in epoxy resins is poorly understood, with little consensus in the published literature. Indeed, it is not unreasonable that the relative importance of the different processes considered above may depend on material factors. As such, we do not believe that speculating on the interpretation of the behaviour seen in Figure 8 and Figure 9 is of significant value. Nevertheless, these results do indicate that the onset of the DC conduction process that dominates at higher temperatures (i.e., for *T* > *T_g_*) is strongly coupled to the molecular motions associated with the dielectric αβ process. This conclusion is consistent with suggestions made elsewhere [46,47] for conventional epoxy resins at a temperature above the relevant *T_g_*.

## 4. Conclusions

A highly flexible amine-cured epoxy resin system has been prepared using DGEBA cured with a long-chain polyoxypropylene diamine hardener (Jeffamine D-4000) in conjunction with an amine-based accelerator. Systems characterised by strain values at the failure of up to ~230% were produced. The molecular dynamics within our DGEBA/D-4000 system is unusual in that only two dielectric relaxation processes are evident, instead of three as reported in relevant studies. At low temperatures (<200 K), a dielectric relaxation process occurs that is commensurate with the γ relaxation, albeit that, the strength/shape of this is very dependent upon temperature, such that conventional quantitative analysis exploiting, for example, time-temperature superposition was not possible. Instead, a quantitative analysis of this parameter was undertaken since it was possible to estimate the frequency corresponding to the local maximum in *ε_r_*″. This showed that the data aligned well with both Arrhenius and activated tunnelling behaviour, with both methodologies giving physically reasonable parameter values. The significance of this analysis is that it further reinforces the assertion of [27] that “pure thermal activation, as described by the Arrhenius equation, is just a manifestation of activated tunnelling above some material dependent temperature”.

In conventional epoxy resins, the γ relaxation is followed at progressively higher temperatures up to *T_g_* by the β and α processes; in our DGEBA/D-4000 system, only one process occurs, which we have therefore termed αβ mode. This relaxation exhibits VFT-like behaviour and, as such, it is akin to the α processes seen in conventional epoxy-based systems. We interpret this behaviour, collectively, as a bifurcation of the αβ relaxation seen here into the α and β processes seen in conventional epoxies, with the implication that the dipolar moieties involved in all three are the same. In the αβ process, as in conventional α relaxation, significant coupling with main chain segments occurs, whereupon VFT behaviour follows.

Finally, charge transport also follows VFT-type behaviour and, significantly, our analysis leads to the same value of *T_VFT_* as derived from the temperature dependence of the αβ process. We, therefore, conclude that charge transport at such temperatures is strongly coupled with the cooperative molecular motions described for the αβ relaxation. 

## Figures and Tables

**Table 1:** Accelerator and hardener compositions.

Accelerator Concentration (phr)	Jeffamine D-4000 Concentration (phr)
0	597
5	562
10	527
12.5	509
15	492
17.5	474
20	457

**Table 2:** Derived Arrhenius and activated tunnelling parameters pertinent to the γ relaxation.

Arrhenius		Activated Tunnelling		
*A*_0_ (THz)	*E_a_* (eV)	Frequency (THz)	Δ (eV)	*d*_0_ (Å)
(5–380) × 10^−3^	0.243 ± 0.024	18.9 ± 0.9	0.322 ± 0.008	2.65 ± 0.09

## Data Availability

Not applicable.
